# Transdifferentiation of periodontal ligament-derived stem cells into retinal ganglion-like cells and its microRNA signature

**DOI:** 10.1038/srep16429

**Published:** 2015-11-09

**Authors:** Tsz Kin Ng, Jasmine S. Y. Yung, Kwong Wai Choy, Di Cao, Christopher K. S. Leung, Herman S. Cheung, Chi Pui Pang

**Affiliations:** 1 Department of Ophthalmology & Visual Sciences, The Chinese University of Hong Kong, Hong Kong; 2 Department of Obstetrics & Gynaecology, The Chinese University of Hong Kong, Hong Kong; 3 Geriatric Research, Education and Clinical Center, Miami Veterans Affairs Medical Center, Miami, FL, United States; 4 Department of Biomedical Engineering, College of Engineering, University of Miami, Coral Gables, FL, United States

## Abstract

Retinal diseases are the leading causes of irreversible visual impairment and blindness in the developed countries. Human retina has limited regenerative power to replace cell loss. Stem cell replacement therapy has been proposed as a viable option. Previously, we have induced human adult periodontal ligament stem cells (PDLSCs) to the retinal lineage. In this study, we modified our induction protocol to direct human adult PDLSCs into retinal ganglion-like cells and determined the microRNA (miRNA) signature of this transdifferentiation process. The differentiated PDLSCs demonstrated the characteristics of functional neurons as they expressed neuronal and retinal ganglion cell markers (ATOH7, POU4F2, β-III tubulin, MAP2, TAU, NEUROD1 and SIX3), formed synapses and showed glutamate-induced calcium responses as well as spontaneous electrical activities. The global miRNA expression profiling identified 44 upregulated and 27 downregulated human miRNAs after retinal induction. Gene ontology analysis of the predicted miRNA target genes confirmed the transdifferentiation is closely related to neuronal differentiation processes. Furthermore, the expressions of 2 miRNA-targeted candidates, VEGF and PTEN, were significantly upregulated during the induction process. This study identified the transdifferentiation process of human adult stem cells into retinal ganglion-like cells and revealed the involvement of both genetic and miRNA regulatory mechanisms.

Glaucoma is one of the leading causes of irreversible visual impairment and blindness in the developed countries^1^. The conventional regimens for glaucoma are based on surgical or medical interventions to reduce intraocular pressure and limit visual loss. However, in many patients, the number of retinal ganglion cells (RGCs) still degenerate progressively irrespective of treatment. Owing to the fact that RGCs and endogenous retinal progenitor cells have limited regenerative power to replace cell loss, new and effective therapies should be developed against these sight-threatening diseases. Recently, the incremental progress in stem cell research has shown promising applications of stem cells in therapeutic treatments.

Currently, there are two main strategies in stem cell therapy: modulating the neuroprotective environment and cell replacement therapy^2^. Neuroprotection relies on provisions of neurotrophic factors and transfer of beneficent molecules. Cell replacement therapy is based on the hypothesis that new RGCs could be generated from stem cells to substitute the damaged cells in the diseased retina. This leads to the establishment of various protocols on the induction of embryonic stem cells (ESCs) into retinal lineage^3^,^4^,^5^,^6^. Similar induction capability also applies to induced pluripotent stem cells (iPSCs)^7^. Retinal induction of adult stem cells have also been demonstrated^8^,^9^. Previously, we reported that the human adult periodontal ligament-derived stem cells (PDLSCs) are capable of differentiating into the neurogenic, cardiomyogenic, chondrogenic and osteogenic lineages^10^. Recently, we successfully induced the PDLSCs to retinal fate, with the PDLSC-derived retinal cells expressing photoreceptor makers^11^. In this study, we modified the induction protocol to generate RGC-like cells with electrophysiological functions. Moreover, we hypothesized that the retinal induction of PDLSCs is governed by genetic and microRNA (miRNA) regulation. Up to date, the gene expression profile of retinal induction has only been reported using ESCs^12^, whereas the miRNA expression profiles only limited to the differentiation of ESCs and iPSCs into retinal pigment epithelial (RPE) cells^13^,^14^,^15^. Nevertheless, miR-125, miR-9 and let-7 have recently been shown to be the key regulators of the retinal progenitor development in mice^16^.

The original retinal induction protocol which we adopted mainly produced Pax6^+^/Chx10^+^ retinal progenitors and RGCs^5^. We, in this study, determined the RGC marker expression, the glutamate-induced calcium response as well as the electrophysiology of the differentiated PDLSCs. Moreover, the miRNA expression profile of retinal induction on human adult PDLSC was identified using microarray platform. In addition, the expression of predicted miRNA targets was also evaluated.

## Results

### Transdifferentiation of human PDLSCs into retinal ganglion-like cells

**Figure 1:**
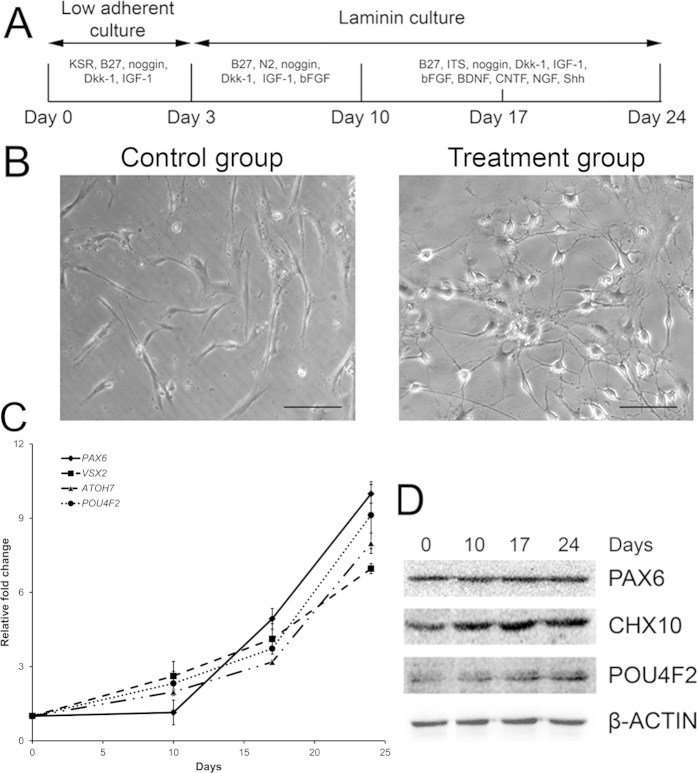
Differentiation of human PDLSCs into retinal ganglion-like cells. (**A**)Human PDLSCs were induced under a modified noggin-Dkk1-IGF1 protocol sequentially by 3 steps for 24 days. RNA and cell lysates were collected at Day 0, 10, 17 and 24. (**B**) The induced PDLSCs showed a neuron-like morphology after induction treatment, whereas the control group showed a fibroblast-like morphology. Scale bar: 100 μm. (**C**) Gene expression analysis for retinal progenitor markers (*PAX6* and *VSX2*) and retinal ganglion cell marker (*ATOH7* and *POU4F2*) by Sybr green PCR. *GAPDH* was used for normalization. (**D**) Protein expression analysis for retinal progenitor markers (PAX6 and CHX10) and retinal ganglion cell marker (POU4F2) on the cell lysates by immunoblotting. β-actin was used as housekeeping protein for normalization.

**Figure 2:**
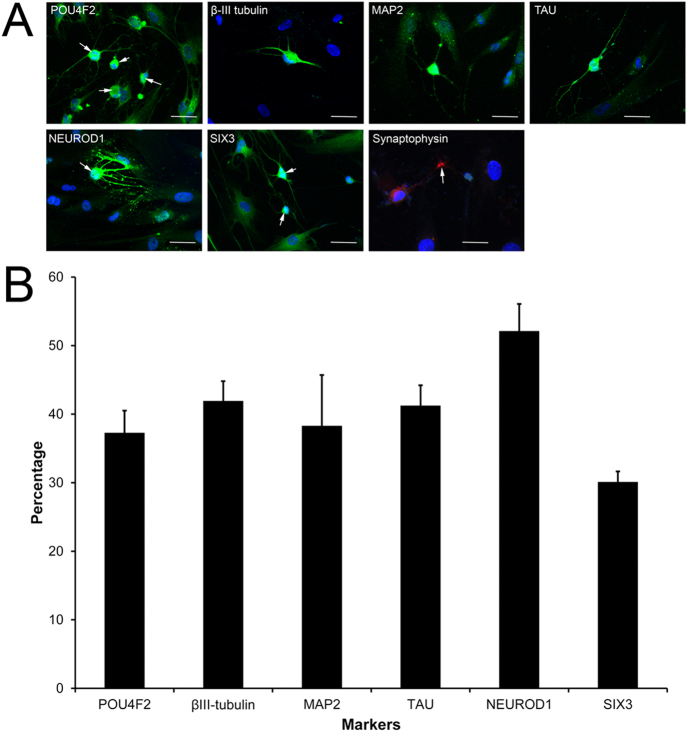
RGC and neuronal marker expression in retinal-induced PDLSCs. The treated cells were fixed at Day 24. (**A**) RGC and neuronal marker expression as well as synapse formation were evaluated by immunofluorescence analysis: β-III tubulin, MAP2, TAU, POU4F2 (nucleus, red arrow), NEUROD1 (nucleus, red arrow), SIX3 (nucleus, red arrow) and synaptophysin. DAPI was used for nuclei counter-stain. Scale bar: 100 μm. (**B**) The frequency of marker expression in the induced PDLSCs.

**Figure 3:**
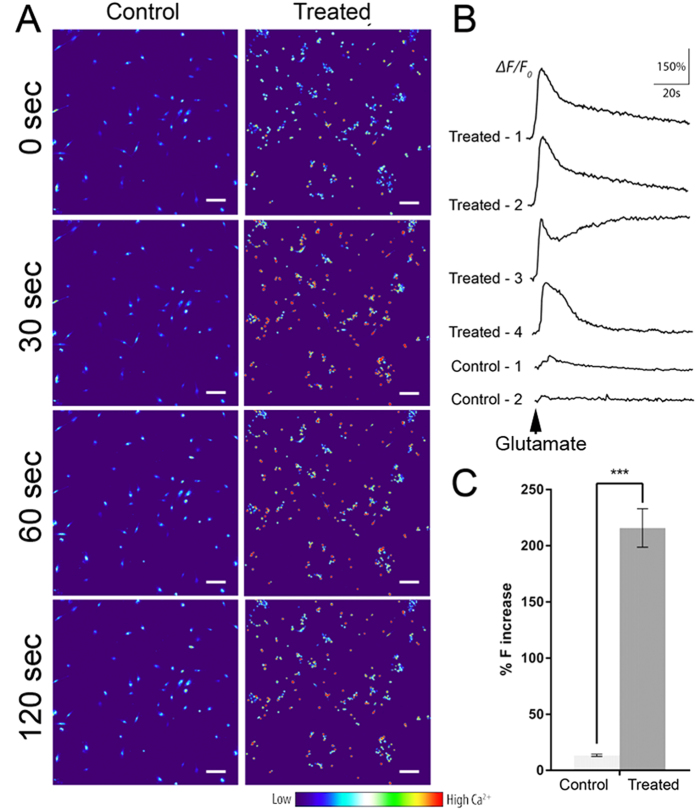
Glutamate-evoked calcium response in retinal-induced PDLSCs. Human PDLSCs were treated under the retinal induction protocol for 24 days. (**A**) Heat map image of Fluo-4AM fluorescence in treated and control PDLSCs after 1 μM glutamate treatment for 30, 60 and 120 seconds. Scale bar: 100 μm. (**B**) Representative traces for spontaneous calcium transient profiles of treated and control PDLSCs. (**C**) Histogram showing the fluorescence intensity changes (%ΔF/F0). Peak calcium responses (mean ± standard deviation) in treated and control PDLSCs were compared to the baseline levels. ***p < 0.001 (paired T-test).

**Figure 4:**
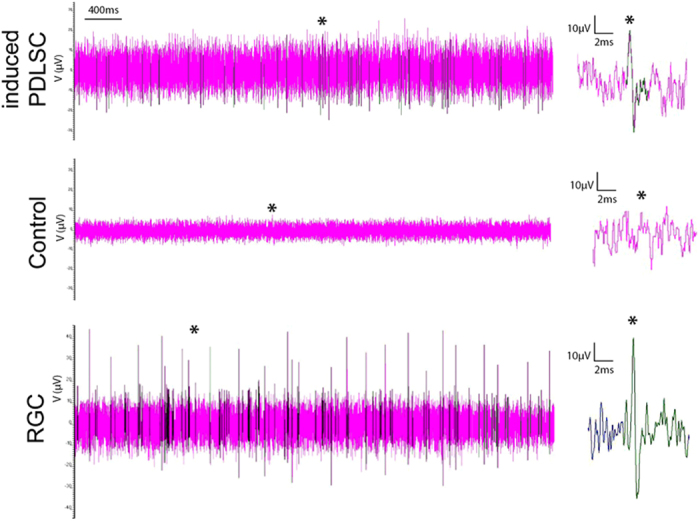
Human PDLSCs demonstrated electrical activity after retinal induction treatment. Human PDLSCs were treated under the retinal induction protocol for 24 days. Spontaneous electrical activities of induced PDLSC, control and purified RGCs were recorded by microelectrode array system. Spikes were defined as the voltage intensity larger than a threshold of 15 μV.

After retinal induction (Fig. 1A), human PDLSCs showed a neuron-like morphology when compared to the fibroblast-like morphology in control group (Fig. 1B). During the retinal induction process of PDLSC, gene expression analysis showed time-dependent upregulation of retinal progenitor markers (*PAX6* and *VSX2*) as well as RGC marker (*ATOH7* and *POU4F2*) when compared to Day 0 undifferentiated PDLSC (Fig. 1C). Similarly, retinal-induced PDLSC highly expressed retinal progenitor marker protein (PAX6 and CHX10) and mature RGC marker protein (POU4F2) after the retinal induction treatment (Fig. 1D). Moreover, immunofluorescence signals of β-III tubulin, MAP2, TAU, POU4F2, NEUROD1 and SIX3 were also detected in the induced PDLSCs (Fig. 2A). 30–52% (mean = 40%) of the induced cells displayed the expression of these retinal and neuronal markers (Fig. 2B). The induced PDLSCs could form synapses with neighboring cells as shown by the immunofluorescence signal of synaptophysin (Fig. 2A). In addition, the induced PDLSCs responded to 1 μM glutamate exposure and showed elevated spontaneous calcium transients when compared to the control group in spike amplitude and intracellular calcium concentration (Fig. 3). Furthermore, the induced PDLSCs could generate spontaneous spikes (Fig. 4). The spike signal of the induced PDLSC was 21.24 ± 1.92 μV with a frequency of 11 spikes per second, whereas that of purified neonatal rat RGCs was 39.27 ± 6.69 μV with a frequency of 17 spikes per second. These electrical signals were not observed in the control group (peak signal = 9.58 ± 1.62 μV), which was close to the background signal (7.07 ± 1.62 μV; data not shown). These data suggested that our induced PDLSCs demonstrated functional neuronal activity. The transdifferentiation of PDLSCs were specific to the neuronal lineage as they were not induced to bone or adipose lineages by our retinal induction treatment, shown by the negative staining of alkaline phosphatase and Oil Red O, respectively (Supplementary Figure 1). Collectively, these indicated that our newly modified noggin-Dkk1-IGF1 induction treatment can direct human PDLSCs into retinal ganglion-like cells, which also confirmed the retinal induction results from our previous report^11^.

### The microRNA signatures of retinal-induced human PDLSCs

**Figure 5:**
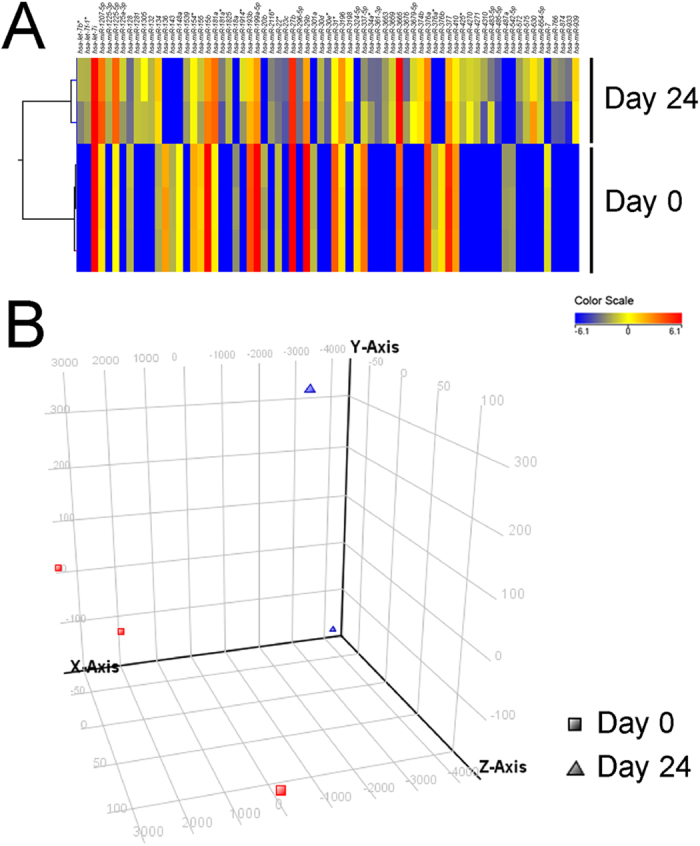
Discrimination of the microRNA microarray profiles in PDLSC before and after retinal induction treatment. (**A**) Supervised hierarchical clustering of the groups before and after retinal induction treatment using the 71 significant human miRNAs identified by microarray. Red color indicates high expression and blue color indicates low expression. (**B**) Principle component analysis of the groups before and after retinal induction treatment using 1205 human miRNAs in the microarray. Rectangle is the group before treatment and triangle is the group after treatment.

**Figure 6:**
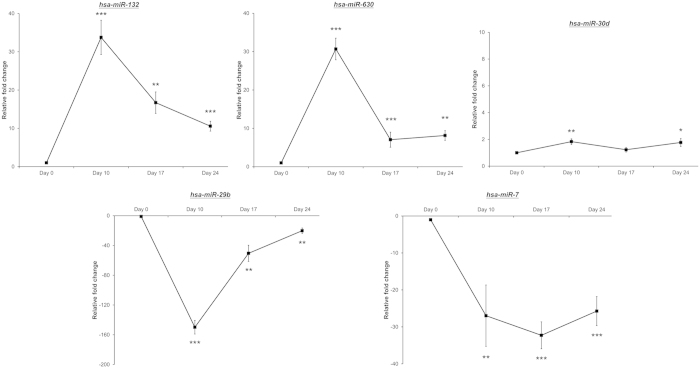
microRNA expression analysis of human PDLSC along the retinal induction treatment. Total RNA was collected at Day 0, 10, 17 and 24. Five significant miRNAs from the microarray profile (*hsa-miR-132*, *hsa-miR-29b*, *hsa-miR-30d*, *hsa-miR-630* and *hsa-miR-7*) were validated using TaqMan PCR approach. snRNA U6 was used for normalization. The relative fold changes were compared to the group at Day 0. The data represented, mean ± standard deviation. *p < 0.05, **p < 0.01, ***p < 0.001.

To understand the molecular mechanism of retinal induction effect, global miRNA expression profile (miRNome) of retinal-induced PDLSC was determined by microarray. The miRNA expression profiles of groups at Day 0 and Day 24 were differentially clustered and separated from each other by either hierarchical clustering (Fig. 5A) or principle component analysis (Fig. 5B). A total of 170 miRNAs were expressed at a two-fold difference in treated PDLSC at Day 24 compared to Day 0. Among these 170 miRNAs, 71 of them were differentially expressed (*p*_*corr*_ < 0.05 and fold change >2; Table 1). 44 of them were upregulated, and 27 of them were downregulated. Based on expression levels, the predicted miRNA target genes and reported neuron-related miRNA^17^,^18^, 5 miRNAs (*hsa-miR-132*, *hsa-miR-29b*, *hsa-miR-30d*, *hsa-miR-630* and *hsa-miR-7*) were selected for validation. *hsa-miR-29b* and *hsa-miR-7* were downregulated throughout the treatment period (Fig. 6). In contrast, *hsa-miR-132* and *hsa-miR-630* were upregulated along the retinal induction treatment. These 4 miRNAs showed significant difference during the treatment period when compared to Day 0. However, *hsa-miR-30d* did not show significant fold-change differences. Therefore, 4 out of 5 selected miRNAs were validated. Notably, comparing to the reported miRNAs in eye and retinal progenitor development, RPE differentiation and retinal degeneration^13^,^14^,^15^,^16^,^19^,^20^, 12 miRNAs showed *p*_*corr*_ < 0.05 (hsa-let-7b^*^, *hsa-let-7d*, *hsa-let-7e*, hsa-let-7f-1^*^, *hsa-let-7i*, *hsa-miR-125a-3p*, *hsa-miR-125a-5p*, *hsa-miR-15b*, *hsa-miR-181a*, *hsa-miR-18a*, *hsa-miR-214*, and *hsa-miR-301a*; Table 2). Subsequently, a global target gene list of the retinal-induced miRNAs (2043 genes) was generated by the TargetScan in the GeneSpring (Agilent) platform. Gene ontology of this gene list, analyzed by DAVID (1942 DAVID identities), revealed that retinal-induced miRNAs might target the genes involved in different processes of neuron differentiation (Table 3).

### microRNA target gene analysis in retinal-induced human PDLSCs

**Figure 7:**
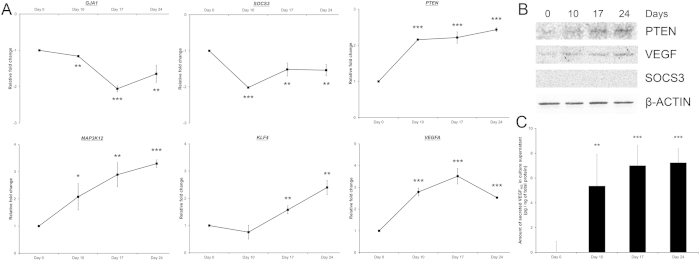
Target gene expression analysis of human PDLSCs along the retinal induction treatment. Human PDLSCs were treated under the retinal induction protocol for 24 days. RNA, cell lysates and culture supernatant were collected at Day 0, 10, 17 and 24. (**A**) Gene expression analysis for neuronal differentiation (*GJA1*), retinogenesis (*KLF4* and *VEGFA*) and retinal ganglion cell markers (*MAP3K12*, *PTEN* and *SOCS3*) by Sybr green PCR. *GAPDH* was used for normalization. The relative fold changes were compared to the group at Day 0. The data represented was the mean ± standard deviation. (**B**) Expression analysis for PTEN, VEGF and SOCS3 protein in the cell lysates by immunoblotting. β-actin was used as housekeeping protein for normalization. (**C**) ELISA analysis for VEGF_165_ secretion in cell culture supernatant. *p < 0.05, **p < 0.01, ***p < 0.001.

From the predicted target gene list, *GJA1*, *KLF4*, *MAP3K12*, *PTEN*, *SOCS3* and *VEGFA* were associated with eye development (Supplementary Table 3) or neuron differentiation (Supplementary Table 4). Therefore, genes for neuronal differentiation (*GJA1*), retinogenesis (*KLF4* and *VEGFA*) and RGC axon regeneration (*MAP3K12*, *PTEN* and *SOCS3*)^21^,^22^ were selected for subsequent gene expression analysis by Sybr green PCR. *GJA1* and *SOCS3* were significantly downregulated in the retinal-induced PDLSCs (Fig. 7A). In contrast, *MAP3K12*, *PTEN*, *KLF4* and *VEGFA* were upregulated along the retinal induction period, compared to the group at Day 0. Coherent to the gene expression result, the expression of PTEN and VEGF protein were elevated during the retinal induction treatment although SOCS3 could not be detected in the PDLSCs (Fig. 7B). In addition, VEGF_165_ secreted by PDLSCs was also significantly increased after retinal induction treatment (Fig. 7C). The gene and protein expression results suggested that genetic regulation also contributes to the retinal induction of human PDLSCs.

### microRNA-132 in retinal progenitor cell differentiation and its target gene analysis in human PDLSCs

**Figure 8:**
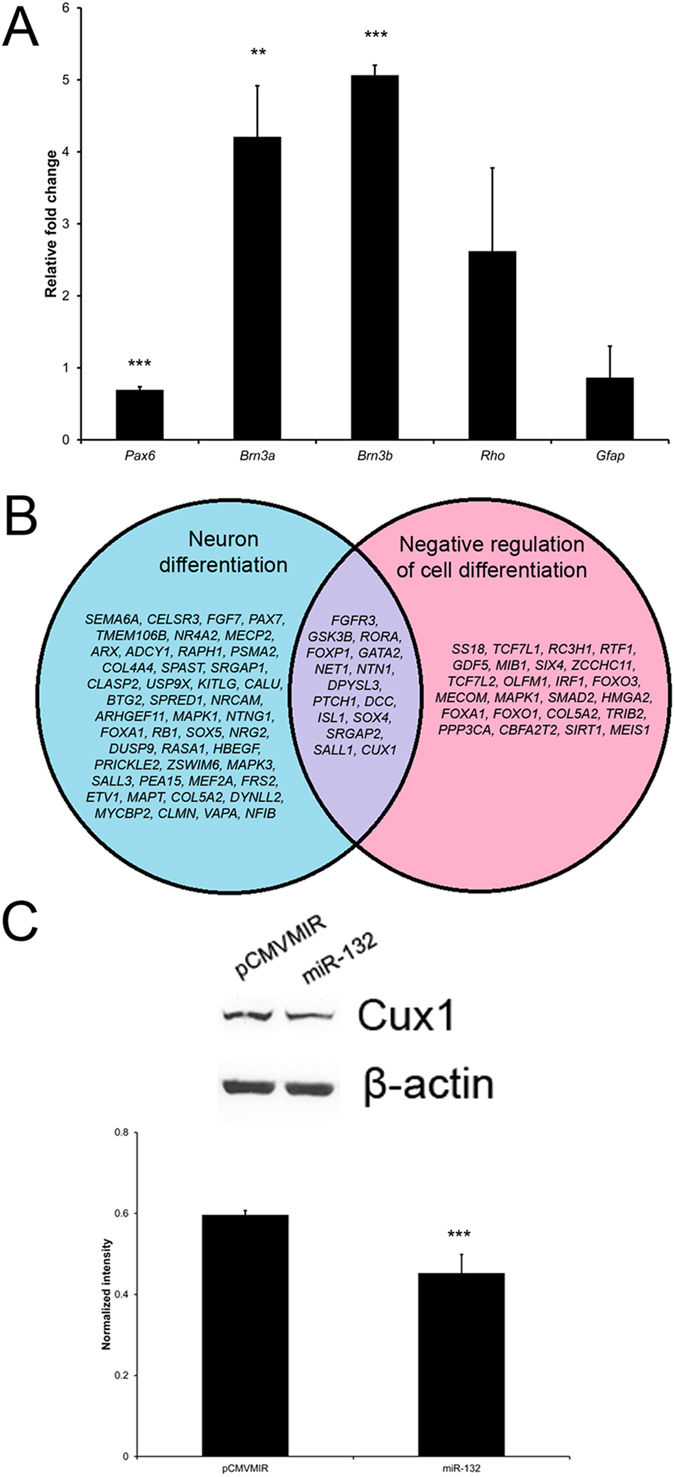
microRNA-132 in retinal progenitor cell differentiation and its target gene analysis in human PDLSCs. (**A**) Expression analysis of retinal marker genes on the miR-132-transfected retinal progenitor cells after differentiation by Sybr green PCR. The expression level was normalized by the housekeeping gene (*Gapdh*) and compared to that of the pCMVMIR-transfected cells. (**B**) Venn diagram of TargetScan-predicted miR-132 target genes related to neuron differentiation and negative regulation of cell differentiation. (**C**) Expression analysis of CUX1 protein on the miR-132-transfected human PDLSCs by immunoblotting. β-actin was used as housekeeping protein for normalization, and the pCMVMIR-transfected cells were used as control. **p < 0.01, ***p < 0.001.

In order to delineate the biological roles of the differentially expressed miRNAs in retinal differentiation process, retinal progenitor cells from embryonic day 18 rats were transfected with an expression plasmid inserted with miR-132 precursor sequence and treated with differentiation medium. Compared to the empty vector control (pCMVMIR), downregulation of *Pax6* gene (1.5 folds, *p* < 0.001) and upregulation of *Brn3a* and *Brn3b* genes (4.2 folds, *p* < 0.01 and 5.1 folds, *p* < 0.001, respectively) were observed in retinal progenitor cells transfected with miR-132 expression plasmid (Fig. 8A). *Rho* and *Gfap* genes showed no significant difference between empty vector control and miR-132-transfected cells. The retinal progenitor cell differentiation analysis suggested that miR-132 could direct retinal progenitor cells towards RGC lineage.

Meanwhile, we performed the gene ontology analysis on the TargetScan-predicted miR-132 target genes (407 genes), and compared the gene lists related to neuron differentiation (62 genes) and negative regulation of cell differentiation (38 genes; Fig. 8B). There are 15 genes in common, which are *FGFR3*, *GSK3B*, *RORA*, *FOXP1*, *GATA2*, *NET1*, *NTN1*, *DPYSL3*, *PTCH1*, *DCC*, *ISL1*, *SOX4*, *SRGAP2*, *SALL1* and *CUX1*. Among these 15 genes, GSK3B, *NET1*, *DPYSL3*, *SOX4*, *SRGAP2*, and *CUX1* showed moderate to strong expression in human PDLSCs, whereas *RORA*, *GATA2* and *SALL1* showed weak expression (data not shown). When human PDLSCs were transfected with miR-132 expression plasmid, the expression of CUX1 (cut-like homeobox 1) protein was reduced by 33% (*p* < 0.001; Fig. 8C), suggesting that *CUX1* gene could be a downstream target gene of miR-132.

## Discussion

Stem cell therapy for retinal diseases has entered the stage of clinical trials. Autologous bone marrow-derived mononuclear cells have been applied in patients with retinitis pigmentosa or cone-rod dystrophy^23^. Moreover, human ESC-derived retinal pigment epithelial cells were used to treat patients with Stargardt’s macular dystrophy and advanced non-exudative age-related macular degeneration by cell replacement therapy^24^. But adult stem cell-derived retinal cells have not been attempted for use in cell replacement therapy. We aim to study human adult stem cells as they can be conveniently obtained from patients for autologous transplantation or *in vitro* drug screening, which is the basis of personalized medicine in future. We made a progress in this direction when we induced human adult PDLSCs to retinal fate^11^. Results of this present study confirm the feasibility of retinal induction on human PDLSCs. We found PDLSCs underwent the noggin-Dkk1-IGF1 induction treatment not only produced photoreceptor cells as we have previously reported^11^, but also expressed RGC and neuronal markers (ATOH7, POU4F2, β-III tubulin, MAP2 and TAU; Figs 1 and 2), formed synapses, gave glutamate-induced calcium responses (Fig. 3) and demonstrated spontaneous electrical activities (Fig. 4) in this study. The mechanism of this retinal induction process warrants further investigation.

This study also, for the first time, reported the miRNA signatures of retinal induction on human adult stem cells. After 24-day retinal induction treatment, 71 human miRNAs were differentially expressed, which 44 of them were upregulated and 27 were downregulated (Table 1). Comparing to the reported miRNAs related to eye and retinal progenitor development, RPE differentiation and retinal degeneration^13^,^14^,^15^,^16^,^19^,^20^, 12 miRNAs showed *p*_*corr*_ < 0.05 (Table 2). Six of them (*hsa-let-7f-1*, *hsa-let-7i*, *hsa-miR-125a*, *hsa-miR-15b*, *hsa-miR-18a* and *hsa-miR-301a*) were commonly found in the differentiation treatment of ESC into retinal pigment epithelial cells^13^. Moreover, let-7 and miR-125 families are key regulators of retinal progenitor cell development^16^. In addition, 17 miRNAs (miR-136, miR-143, miR-148a, miR-15b, miR-18a, miR-181a, miR-181a*, miR-20b, miR-27b, miR-29b, miR-30d, miR-30e*, miR-301a, miR-376a, miR-376b, miR-410 and miR-7), which are differentially expressed in our retinal induction treatment, are involved in the regulation of developing mouse retina^25^. Thus our miRNA profile is closely related to the retinal cells formation processes, indicating that the commonly found miRNAs may guide human PDLSCs to retinal precursors with a similar differentiation pathway as ESC and retinal development in mice.

Among the upregulated miRNAs, miR-132 is expressed in RGCs^26^ and regulates neuron development and neurite morphology^17^,^18^. In this study, we, for the first time, showed that miR-132 could be involved in the RGC differentiation from retinal progenitor cells as its overexpression in retinal progenitor cells upregulates mature RGC markers (*Brn3a* and *Brn3b*) expression (Fig. 8A). This further confirms that miR-132 contributes to the differentiation of human PDLSCs into retinal ganglion-like cells. Moreover, we also demonstrated that miR-132 could downregulate CUX1 protein expression in human PDLSCs (Fig. 8C). Since reduction in Cux1 expression has been shown to promote neurite outgrowth in cortical neurons^27^, the morphology of human PDLSC-derived neurons could be related to the interactive functions of miR-132 and *CUX1* in our trans-differentiation process. In addition, the properties of miR-132 are consistent with the gene ontology analysis of the predicted miRNA target genes (Table 3). Furthermore, BDNF and bFGF were suggested to induce miR-132 expression^28^,^29^, indicating that BDNF and bFGF in our treatment medium might be responsible for the upregulation of miR-132 in the induced PDLSCs. Notably, the highest upregulated miRNA, *hsa-miR-630*, was correlated with cancer cell death^30^,^31^. This is the first report that it could be associated with neuronal differentiation of human adult stem cells. In addition, *GJA1*, which is a negative modulator of neuronal differentiation^32^, was downregulated along the retinal induction period (Fig. 7), further affirming our PDLSC proceed towards neuronal differentiation under the retinal induction treatment.

We chose the 5 miRNAs (*hsa-miR-132*, *hsa-miR-29b*, *hsa-miR-30d*, *hsa-miR-630* and *hsa-miR-7*) for validation not just based on the previous reports or the expression levels, but also the predicted target genes. *VEGFA* is the target gene of *hsa-miR-29b* and *hsa-miR-15b* (Supplementary Table 2), which were downregulated during the retinal induction treatment (Table 1 and Fig. 6). From miRNA target expression analyses, we found that *VEGFA* gene expression, VEGF protein expression and VEGF_165_ secretion were all increased (Fig. 7). These findings verify the prediction that *VEGFA* upregulation could be correlated with *hsa-miR-29b* and *hsa-miR-15b* downregulation. Furthermore, hypoxia, which upregulates VEGF, has been shown to increase retinogenesis from ESC^33^. Therefore, the elevated VEGF level, together with downregulation of *hsa-miR-29b* and *hsa-miR-15b*, should account for retinal differentiation of human adult stem cells.

Our retinal induction protocol produces retinal ganglion-like cells (Figs 1, 2, 3, 4). The selected target genes (*KLF4*, *MAP3K12*, *PTEN* and *SOCS3*) are closely related to RGCs for gene expression analysis^21^,^22^,^34^. *MAP3K12*, *PTEN* and *SOCS3* genes as well as the PTEN protein were upregulated during the retinal induction process (Fig. 7). Since deletion of Pten at the onset of neurogenesis in retinal progenitor cells results in the reduction of RGCs and rod photoreceptors^35^, our results further suggest that the treated PDLSC undergo proper retinal/neuronal differentiation guided by PTEN regulation. Moreover, *KLF4*, the predicted target of *hsa-miR-7* (Supplementary Table 3), was upregulated during the retinal induction treatment (Fig. 7). Accordingly, *KLF4* could also participate in RGC development during PDLSC retinal differentiation^34^. Nevertheless, we predicted that *KLF4* upregulation could be correlated with *hsa-miR-7* downregulation (Figs 6 and 7), which is confirmed by a recent study on neural stem cell differentiation^36^. In-depth functional analyses as well as the relationship with putative target genes are needed to validate the role of miRNAs in retinal induction process of human adult stem cells.

In summary, this study, for the first time, demonstrated transdifferentiation of human PDLSCs into functional retinal ganglion-like cells, and identified miR-132, VEGF and PTEN as key regulators in the retinal fate determination of human adult stem cells. Results from this study reveal the involvements of both genetics and miRNA regulatory mechanisms in human adult stem cell retinal differentiation.

## Methods

### Human periodontal ligament-derived stem cell culture

Human PDLSC lines were established previously^10^,^11^. These cells were cultured in Dulbecco’s modified Eagle’s medium (high glucose; Gibco BRL, Rockville, MD) supplemented with 10% heat-inactivated fetal bovine serum (FBS; Gibco BRL). Cells with passage 3–5 were used for the retinal induction experiments.

### Retinal induction

Human PDLSCs were induced by a previously established retinal induction protocol^5^,^11^ with modification (Fig. 1A). Briefly, PDLSCs (200 cells/μl) were first treated with 10% knockout serum replacement (Gibco BRL), 1x B27 supplement (Gibco BRL), 1 ng/ml noggin (PeproTech, Rocky Hill, NJ), 1 ng/ml dickkopf-related protein 1 (Dkk-1; PeproTech) and 5 ng/ml insulin-like growth factor 1 (IGF-1; PeproTech) in Dulbecco’s modified Eagle’s medium: nutrient mixture F-12 (DMEM/F12; Gibco BRL) at low adherent culture for 3 days. After 3 days, the cell aggregates were transferred to the dishes coated with laminin (Sigma-Aldrich, St. Louis, MO) and treated with 1x B27 supplement, 1x N2 supplement (Gibco BRL), 100 ng/ml noggin, 10 ng/ml Dkk-1, 100 ng/ml IGF-1 and 50 ng/ml basic fibroblast growth factor (bFGF; PeproTech) in DMEM/F12 medium for 7 days. The culture medium was then switched to 1x B27 supplement, 1x insulin-transferin-selenium supplement (Gibco BRL), 10 ng/ml noggin, 10 ng/ml Dkk-1, 10 ng/ml IGF-1, 50 ng/ml bFGF, 10 ng/ml brain-derived neurotrophic factor (BDNF; PeproTech), 10 ng/ml ciliary neurotrophic factor (CNTF; PeproTech), 10 ng/ml nerve growth factor (NGF; PeproTech) and 10 ng/ml sonic hedgehog (Shh; PeproTech) in DMEM/F12 medium and further treated for 14 days. Culture medium was changed every 3 days. The control medium was the DMEM/F12 medium supplemented with 10% FBS. Culture supernatant, total RNA and cell extracts from the culture cells were collected at Day 0, 10, 17 and 24 for further analyses.

### Glutamate-evoked calcium response

Spontaneous intracellular calcium transient was evaluated using fluo-4-acetoxymethyl ester (Fluo-4AM; Invitrogen)^11^. Briefly, the treated or control PDLSCs at Day 24 were incubated in Hanks’ balanced salt solution (HBSS, Ca^2+^/Mg^2+^-free; Gibco BRL) containing 5μM Fluo-4AM and 0.1% pluronic F-127 (Sigma-Aldrich) for 30 minutes at room temperature. After washing with HBSS, 1 μM glutamate (Sigma-Aldrich) was added to the culture, and fluorescence images were immediately captured using a fluorescence microscope (Eclipse Ni-C; Nikon, Japan), with excitation wavelength at 495 nm and emission wavelength at 515 nm, every 5 seconds for a total of 2 minutes under a 40X objective lens. Fluorescence intensity at specific time intervals was measured on a total of 50 cells in triplicate experiments (at least 10 cells in each sample) by the NIS-Element AR software (Nikon). The cellular change of fluorescence (F; %ΔF/F_baseline_) of each region was calculated as (F_treated_ – F_baseline_)/F_baseline_ X 100%. The Ca^2+^ fluorescence ratio was converted into Ca^2+^ concentration using the equation [Ca^2+^]_i_ = K*R*/{K/([Ca^2+^]_rest_ + 1) − *R*}^37^, where K is the dissociation constant of Fluo-4AM (400 nM), *R* is the fluorescence ratio (ΔF/F_baseline_) and [Ca^2+^]_rest_ is the resting Ca^2+^ concentration (100 nM in neuronal cells)^38^.

### Electrical activity analysis

Spontaneous electrical activities of the induced PDLSCs were detected by a microelectrode array (MEA) system (Multi Channel Systems, Reutlingen, Germany) on a laminin-coated 54-electrode 6-well MEA chip at Day 24 of differentiation. The differentiated cells were seeded onto the MEA chip at least 7 days prior to recording. The MEA signals, sampled at 25 mHz for 200 seconds, were amplified and digitized using the MEA-2100-60 system (Multi Channel Systems) with integrated temperature controller, data acquisition interface. The spike events were then filtered and analyzed using the MCRack software (Multi Channel Systems). The field potentials where electrodes exhibiting spike frequencies below 0.1 Hz were removed by a 0.1 Hz High Pass filter based on reported minimal steady firing rates^39^. Spikes were detected after spike sorting at a threshold of 15 μV^40^. The MEA signal of purified neonatal RGCs was used as a reference.

### microRNA microarray and data analysis

The protocol for microRNA microarray analysis has been established previously^41^. Briefly, total RNA, including the miRNA fraction, in TRIzol reagent was extracted according to the manufacturer’s protocol (Invitrogen; Carlsbad, CA). The RNA concentration and quality were measured by Nanodrop 2000, whereas the RNA integrity was determined by the Agilent 2100 Bioanalyzer. The SurePrint G3 Human v16 miRNA Array Kit (8 × 60K, Release 16.0; Agilent, Foster City, CA) containing probes for 1205 human and 144 human viral miRNAs from the Sanger miRBase v16.0 was used. GeneSpring GX 11.5 software (Agilent) was used for value extraction. A 2-tailed Student’s t-test was then used for the calculation of the *p*-value for each miRNA probe. Significance was defined by the fold change greater than 2 and corrected *p*-value less than 0.05. Benjamini-Hochberg false discovery rate was used for the multiple testing correction. Principal component analysis and hierarchical clustering were performed to provide a visual impression of how various sample groups are related. Three samples at Day 0 and 2 samples at Day 24 were used in the miRNA microarray experiment.

Downstream mRNA targets of the miRNAs were predicted by TargetScan (http://genes.mit.edu/targetscan/index.html) in the GeneSpring GX 11.5 software (Agilent). Context percentile of 95 was used as the criteria for target prediction. Gene enrichment and gene ontology analysis was performed by DAVID Bioinformatics Resources 6.7 (http://david.abcc.ncifcrf.gov/)^42^. Enrichment score greater than 1.3 was considered as significant. Based on expression levels, the predicted miRNA target genes and reported neuron-related miRNA^17^,^18^, 5 miRNAs from microarray results (*hsa-miR-132*, *hsa-miR-29b*, *hsa-miR-30d*, *hsa-miR-630* and *hsa-miR-7*) were selected for validation. Total RNA (20 ng) was reverse transcribed using the TaqMan MicroRNA Reverse Transcriptase kit (Applied Biosystems, Forster City, CA). The resultant products were quantified using the appropriate TaqMan MicroRNA Assays (Applied Biosystems) on a Stratagene Mx3005P Real-Time PCR Detection System (Stratagene, La Jolla, CA). Results were all normalized to U6 expression. Independent T-test was used for statistical analysis. Three independent samples from each time-point were used in the validation experiment.

### Gene and protein expression analyses

To validate our retinal induction on PDLSC producing RGCs, retinal progenitor (PAX6, VSX2/Chx10), RGC (ATOH7 and POU4F2) and neuronal (TAU, MAP2, β-III tubulin, NEUROD1 and SIX3) marker expression as well as synapse formation analysis (synaptophysin) were performed by semi-quantitative PCR with specific primers (Supplementary Table 1), immunoblotting or immunofluorescence analysis with specific antibodies (Supplementary Table 2). The predicted miRNA target genes related to eye development or neuron differentiation (GJA1, KLF4, MAP3K12, PTEN, SOCS3 and VEGFA) were also examined.

For gene expression analysis of the predicted miRNA target genes, Sybr green PCR (Applied Biosystems) was performed on a real-time PCR machine (Stratagene, La Jolla, CA). Housekeeping gene (*GAPDH*) was used for normalization. The relative expression levels were compared to that of Day 0.

For immunoblotting analysis, the induced PDLSCs were lysed by RIPA buffer supplemented with protease and phosphatase inhibitors. The total protein concentrations of the cell lysates were measured by Protein assay (BioRad, Hercules, CA). Equal amount of total protein (10 μg) for each denatured samples were resolved on 12.5% SDS-polyacrylamide gel and electro-transferred to nitrocellulose membranes for sequential probing with the primary antibodies and secondary antibodies conjugated with horseradish peroxidase (Santa Cruz Biotechnology, Santa Cruz, CA). The signals were detected by enhanced chemiluminescence (Amersham Pharmacia, Cleveland, OH) with the ChemiDoc^TM^ XRS^+^ system (BioRad). β-actin was used as housekeeping protein for normalization.

For immunofluorescence analysis, the induced PDLSCs were fixed in 4% paraformaldehyde (Sigma Aldrich, St. Louis, MO). After permeation and blocking, the cells were labeled with different primary antibodies against RGC or neuronal markers and secondary antibodies conjugated with Alexa Fluor®488 or 594 (Santa Cruz Biotechnology). DAPI was used for the nuclear staining. The fluorescence signals were visualized under a fluorescence microscope (Eclipse Ni-U; Nikon).

VEGF_165_ secretion was analyzed by ELISA assay. Briefly, culture supernatant was concentrated by a 3-kDa centrifugal filtration unit (Millipore, Billerica, MA), and total protein concentration was measured by Protein assay (BioRad). Equal amount of total protein (1 ng) was applied to the human VEGF Quantikine^®^ ELISA assay (R&D Systems Inc., Minneapolis, MN) according to the manufacturer’s protocol. Absorbance at 450 nm with a reference of 540 nm was measured by a spectrophotometer (Powerwave XS, Bio-Tek Instruments). The amount of VEGF_165_ secreted to the culture supernatant (pg of VEGF_165_/ng of total protein in culture supernatant) were then determined.

### Retinal progenitor cell differentiation

Retinas from embryonic day 18 Sprague Dawley rats were digested with 0.05% trypsin (Gibco BRL) for 5 min. The retinal cells were dissociated by repeat pipetting in 1 mg/ml trypsin inhibitor (Sigma-Aldrich). Single cells were collected by passing through the 40 μm cell strainer and cultured in the low adherent dish with Neurobasal A medium (Gibco BRL) supplemented with 1 × N2 (Gibco BRL), 1% bovine serum albumin (Sigma-Aldrich), 20 nM progesterone (Sigma-Aldrich), 20 ng/ml bFGF (PeproTech), 30 ng/ml epidermal growth factor (EGF; PeproTech) and 2 μg/ml heparin (Sigma-Aldrich) for 3 days. Neurospheres were collected, transfected with miR-132 expression plasmid (OriGene, Rockville, MD) and cultured in the differentiation medium (Neurobasal A medium with 1x B27) for 7 days. RNA of the differentiated cells was collected and isolated as previously mentioned. Expression analysis on *Pax6*, *Brn3a*, *Brn3b*, *Rho* and *Gfap* genes was performed by Sybr green PCR with specific primers (Supplementary Table 1). The expression level was normalized by the housekeeping gene (*Gapdh*) and compared to that of the empty vector (pCMVMIR)-transfected cells. All rats were treated according to the guidelines of the ARVO Statement for the Use of Animals in Ophthalmic and Vision Research. The experimental protocol was approved by the Animal Experimentation Ethics Committee of the Chinese University of Hong Kong.

### microRNA-132 transfection analysis

The miR-132 expression plasmid based on the PCMVMIR cloning vector was purchased from the commercially available source (OriGene). The miR-132 expression plasmid was transfected into human PDLSCs through the TransIT®-LT1 transfection reagent (Mirus Bio LLC, Madison, WI) according to the manufacturer’s protocol. Five days after transfection, protein from the transfected cell was collected for CUX1 expression analysis by immunoblotting as previously mentioned. β-actin was used as housekeeping protein for normalization. The expression level of the miR-132-transfected cells was compared to that of pCMVmiR-transfected cells.

### Statistical analysis

All statistical analyses, except microarray analysis, were performed by commercially available software (SPSS, version 20.0; SPSS Inc., Chicago, IL). Independent T-test was used to compare the means between samples. Significance was defined as *p* < 0.05.

## Additional Information

**How to cite this article**: Ng, T.K. *et al*. Transdifferentiation of periodontal ligament-derived stem cells into retinal ganglion-like cells and its microRNA signature. *Sci. Rep.* **5**, 16429; doi: 10.1038/srep16429 (2015).

## Supplementary Material

Supplementary Information

## Figures and Tables

**Table 1:** Differentially expressed microRNAs in PDLSC before and after retinal induction treatment.

microRNA	Fold	*p*_*corr*_
*hsa-miR-630*	+276.49	5.81 × 10^−3^
*hsa-miR-374b*	+201.83	2.96 × 10^−4^
*hsa-miR-939*	+201.72	5.47 × 10^−4^
*hsa-miR-3679-5p*	+119.09	2.96 × 10^−3^
*hsa-miR-3198*	+116.80	6.48 × 10^−3^
*hsa-miR-4270*	+107.72	1.47 × 10^−3^
*hsa-miR-1305*	+103.75	7.49 × 10^−3^
*hsa-miR-3659*	+94.36	3.49 × 10^−4^
*hsa-miR-654-5p*	+91.76	2.96 × 10^−4^
*hsa-miR-4271*	+79.82	3.66 × 10^−4^
*hsa-miR-1281*	+79.21	2.96 × 10^−4^
*hsa-miR-575*	+78.37	4.09 × 10^−4^
hsa-miR-425*	+75.96	5.47 × 10^−4^
*hsa-miR-1825*	+73.26	2.96 × 10^−4^
hsa-miR-1914*	+72.55	1.08 × 10^−2^
*hsa-miR-132*	+69.79	2.96 × 10^−4^
*hsa-miR-125a-3p*	+69.41	5.68 × 10^−3^
*hsa-miR-1225-3p*	+68.42	5.54 × 10^−4^
*hsa-miR-30d*	+64.37	5.47 × 10^−4^
*hsa-miR-483-5p*	+62.87	2.49 × 10^−2^
hsa-let-7f-1*	+61.05	6.18 × 10^−4^
*hsa-miR-1539*	+58.84	5.47 × 10^−4^
*hsa-miR-3653*	+54.76	3.23 × 10^−4^
hsa-let-7b*	+52.88	5.71 × 10^−3^
hsa-miR-2116*	+47.94	1.25 × 10^−3^
*hsa-miR-572*	+44.61	4.86 × 10^−3^
hsa-miR-34a*	+42.80	5.81 × 10^−3^
*hsa-miR-3676*	+42.77	9.40 × 10^−3^
*hsa-miR-4310*	+40.40	2.50 × 10^−2^
*hsa-miR-766*	+37.30	1.27 × 10^−2^
*hsa-miR-296-5p*	+34.97	3.43 × 10^−4^
*hsa-miR-874*	+34.45	8.39 × 10^−3^
*hsa-miR-361-3p*	+32.62	3.47 × 10^−4^
hsa-miR-181a*	+32.12	5.81 × 10^−3^
hsa-miR-30e*	+29.12	3.64 × 10^−4^
*hsa-miR-23c*	+29.06	7.64 × 10^−4^
*hsa-miR-933*	+24.20	3.51 × 10^−2^
*hsa-miR-485-5p*	+24.19	3.08 × 10^−3^
*hsa-miR-1225-5p*	+4.36	3.29 × 10^−3^
*hsa-miR-181a*	+3.74	2.96 × 10^−3^
*hsa-miR-134*	+3.52	1.27 × 10^−2^
*hsa-miR-1207-5p*	+3.20	4.23 × 10^−2^
*hsa-miR-3665*	+2.81	4.01 × 10^−2^
*hsa-miR-3196*	+2.09	1.45 × 10^−2^
*hsa-miR-136*	−433.60	3.43 × 10^−4^
*hsa-miR-324-5p*	−247.52	1.92 × 10^−4^
*hsa-miR-376b*	−232.58	1.92 × 10^−4^
*hsa-miR-148a*	−171.98	1.92 × 10^−4^
*hsa-miR-7*	−119.99	3.43 × 10^−4^
hsa-miR-376a*	−93.49	7.33 × 10^−4^
*hsa-miR-301a*	−80.47	3.10 × 10^−4^
*hsa-miR-143*	−77.85	2.96 × 10^−4^
*hsa-miR-20b*	−72.13	5.24 × 10^−4^
*hsa-miR-542-5p*	−62.53	2.96 × 10^−4^
*hsa-miR-487a*	−59.21	2.96 × 10^−4^
*hsa-miR-18a*	−57.61	4.81 × 10^−4^
*hsa-miR-29b*	−20.16	5.08 × 10^−3^
*hsa-miR-337-5p*	−8.80	4.01 × 10^−2^
*hsa-miR-15b*	−6.53	1.11 × 10^−2^
*hsa-miR-199a-5p*	−5.27	3.29 × 10^−3^
hsa-miR-31*	−4.66	2.96 × 10^−4^
*hsa-miR-155*	−3.60	3.89 × 10^−2^
*hsa-miR-377*	−3.40	4.73 × 10^−2^
*hsa-miR-193b*	−3.14	4.01 × 10^−2^
*hsa-miR-27b*	−2.28	5.08 × 10^−3^
*hsa-let-7i*	−2.28	5.81 × 10^−3^
hsa-miR-154*	−2.28	3.64 × 10^−2^
*hsa-miR-410*	−2.24	4.23 × 10^−3^
*hsa-miR-128*	−2.21	1.27 × 10^−2^
hsa-miR-22*	−2.12	8.51 × 10^−3^
*hsa-miR-376a*	−2.01	4.23 × 10^−2^

*p*_*corr*_ represents the *p*-value corrected by Benjamini-Hochberg false discovery rate.

**Table 2:** The fold change analysis of microRNAs related to eye and retinal development, differentiation and degeneration.

microRNA	Fold	*p*_*corr*_
*hsa-let-7a*	−1.22	7.54 × 10^−2^
hsa-let-7a*	−1.16	7.54 × 10^−2^
*hsa-let-7b*	+1.02	8.48 × 10^−1^
hsa-let-7b*	+52.88	5.71 × 10^−3^
*hsa-let-7c*	−1.78	7.03 × 10^−2^
hsa-let-7c*	−1.16	7.54 × 10^−2^
*hsa-let-7d*	−1.37	5.81 × 10^−3^
hsa-let-7d*	−1.16	7.54 × 10^−2^
*hsa-let-7e*	+1.44	1.71 × 10^−2^
hsa-let-7e*	−1.16	7.54 × 10^−2^
*hsa-let-7f*	−1.22	7.54 × 10^−2^
hsa-let-7f-1*	+61.05	6.18 × 10^−4^
hsa-let-7f-2*	−1.16	7.54 × 10^−2^
*hsa-let-7g*	+1.26	2.89 × 10^−1^
hsa-let-7g*	−1.16	7.54 × 10^−2^
*hsa-let-7i*	−2.28	5.81 × 10^−3^
hsa-let-7i*	−1.16	7.54 × 10^−2^
*hsa-miR-100*	+1.15	5.70 × 10^−1^
*hsa-miR-106b*	−5.31	7.54 × 10^−2^
*hsa-miR-10a*	−1.16	7.54 × 10^−2^
*hsa-miR-124*	−1.16	7.54 × 10^−2^
*hsa-miR-125a-3p*	+69.41	5.68 × 10^−3^
*hsa-miR-125a-5p*	+1.44	2.66 × 10^−2^
*hsa-miR-125b*	+1.22	2.88 × 10^−1^
hsa-miR-125b-1*	−1.16	7.54 × 10^−2^
hsa-miR-125b-2*	−1.16	7.54 × 10^−2^
*hsa-miR-130a*	−4.02	7.54 × 10^−2^
*hsa-miR-130b*	−3.75	1.00 × 10^−1^
*hsa-miR-1323*	−1.16	7.54 × 10^−2^
*hsa-miR-139-3p*	−1.16	7.54 × 10^−2^
*hsa-miR-139-5p*	−1.16	7.54 × 10^−2^
*hsa-miR-146a*	+25.34	7.54 × 10^−2^
*hsa-miR-151-3p*	+1.23	1.42 × 10^−1^
*hsa-miR-151-5p*	+1.38	1.27 × 10^−1^
*hsa-miR-152*	+1.07	5.54 × 10^−1^
*hsa-miR-15a*	−7.91	7.54 × 10^−2^
*hsa-miR-15b*	−6.53	1.11 × 10^−2^
*hsa-miR-16*	−2.48	7.54 × 10^−2^
hsa-miR-16-1*	−1.16	7.54 × 10^−2^
hsa-miR-16-2*	−1.16	7.54 × 10^−2^
*hsa-miR-17*	−4.50	7.54 × 10^−2^
*hsa-miR-181a*	+3.74	2.96 × 10^−3^
*hsa-miR-181d*	−1.16	7.54 × 10^−2^
*hsa-miR-182*	−1.16	7.54 × 10^−2^
hsa-miR-182*	−1.16	7.54 × 10^−2^
*hsa-miR-183*	−1.16	7.54 × 10^−2^
hsa-miR-183*	−1.16	7.54 × 10^−2^
*hsa-miR-184*	−1.16	7.54 × 10^−2^
*hsa-miR-187*	−1.16	7.54 × 10^−2^
*hsa-miR-18a*	−57.61	4.81 × 10^−4^
*hsa-miR-194*	−1.16	7.54 × 10^−2^
*hsa-miR-203*	−1.16	7.54 × 10^−2^
*hsa-miR-204*	−1.16	7.54 × 10^−2^
*hsa-miR-214*	−1.75	9.40 × 10^−3^
*hsa-miR-22*	−1.20	2.84 × 10^−1^
*hsa-miR-222*	+1.17	5.42 × 10^−1^
*hsa-miR-23a*	+1.08	1.66 × 10^−1^
*hsa-miR-25*	−1.09	7.64 × 10^−1^
*hsa-miR-26a*	+1.75	7.30 × 10^−2^
hsa-miR-26a-1*	−1.16	7.54 × 10^−2^
hsa-miR-26a-2*	−1.16	7.54 × 10^−2^
*hsa-miR-29c*	−2.02	7.96 × 10^−2^
*hsa-miR-301a*	−80.47	3.10 × 10^−4^
*hsa-miR-302a*	−1.16	7.54 × 10^−2^
hsa-miR-302a*	−1.16	7.54 × 10^−2^
*hsa-miR-302b*	−1.16	7.54 × 10^−2^
hsa-miR-302b*	−1.16	7.54 × 10^−2^
*hsa-miR-302c*	−1.16	7.54 × 10^−2^
*hsa-miR-302c*	−1.16	7.54 × 10^−2^
hsa-miR-302c*	−1.16	7.54 × 10^−2^
*hsa-miR-302d*	−1.16	7.54 × 10^−2^
hsa-miR-302d*	−1.16	7.54 × 10^−2^
*hsa-miR-302e*	−1.16	7.54 × 10^−2^
*hsa-miR-302f*	−1.16	7.54 × 10^−2^
*hsa-miR-3120*	−1.16	7.54 × 10^−2^
*hsa-miR-32*	−1.16	7.54 × 10^−2^
*hsa-miR-33a*	−1.16	7.54 × 10^−2^
*hsa-miR-363*	−1.16	7.54 × 10^−2^
*hsa-miR-373*	−1.16	7.54 × 10^−2^
*hsa-miR-374a*	+1.18	3.14 × 10^−2^
*hsa-miR-512-3p*	−1.16	7.54 × 10^−2^
*hsa-miR-512-5p*	−1.16	7.54 × 10^−2^
*hsa-miR-516b*	−1.16	7.54 × 10^−2^
*hsa-miR-548f*	−1.16	7.54 × 10^−2^
*hsa-miR-598*	−1.16	7.54 × 10^−2^
*hsa-miR-9*	−1.16	7.54 × 10^−2^
*hsa-miR-93*	−2.12	7.54 × 10^−2^
*hsa-miR-96*	−1.16	7.54 × 10^−2^
hsa-miR-96*	−1.16	7.54 × 10^−2^
*hsa-miR-98*	−1.84	7.54 × 10^−2^

*p*_*corr*_ represents the *p*-value corrected by Benjamini-Hochberg false discovery rate.

**Table 3:** The gene ontology analysis of the predicted target genes for the microRNAs differentially expressed after retinal induction treatment.

Functional Annotation	Enrichment score	Count	%	*p*
Respiratory system development	7.13	33	1.71	8.58 × 10^−8^
Neuron differentiation	3.81	79	4.09	8.72 × 10^−6^
Blood vessel development	3.7	49	2.54	4.19 × 10^−5^
Cell migration	3.35	52	2.69	1.19 × 10^−4^
Regulation of cell migration	3.33	35	1.81	3.15 × 10^−4^
Extracellular matrix	3.21	56	2.90	2.97 × 10^−4^
Neural tube development	3.05	17	0.88	2.30 × 10^−3^
Cell adhesion	2.75	104	5.39	9.72 × 10^−4^
JNK cascade	2.67	19	0.98	3.66 × 10^−5^
Tube morphogenesis	2.59	32	1.66	1.26 × 10^−5^
Basement membrane	2.59	16	0.83	9.33 × 10^−3^
MAPKKK cascade	2.34	38	1.97	1.77 × 10^−4^
Regulation of synaptic transmission	2.29	27	1.40	3.20 × 10^−3^
Cytoskeleton	2.25	170	8.80	2.00 × 10^−3^
Anterior/posterior pattern formation	2.18	26	1.35	9.29 × 10^−3^
Central nervous system neuron differentiation	2.12	14	0.73	2.16 × 10^−4^
Synapse	2.09	55	2.85	1.05 × 10^−3^
Regulation of Rho protein signal transduction	1.84	20	1.04	1.03 × 10^−2^
Limb development	1.69	21	1.09	7.58 × 10^−3^
Ear development	1.65	18	0.93	2.80 × 10^−2^
Apoptosis	1.59	80	4.14	4.88 × 10^−2^
Dilated cardiomyopathy	1.52	17	0.88	3.13 × 10^−2^
Muscle tissue development	1.49	22	1.14	3.02 × 10^−2^
Neural crest cell differentiation	1.46	10	0.52	7.43 × 10^−3^
Activation of JUN kinase activity	1.46	9	0.47	5.18 × 10^−3^
Response to estrogen stimulus	1.43	19	0.98	3.57 × 10^−2^
Response to oxidative stress	1.34	28	1.45	1.99 × 10^−2^
Regulation of neuron differentiation	1.31	24	1.24	1.76 × 10^−2^

Enrichment score greater than 1.3 was considered as significant.
