# Supplementary material for: Transdifferentiation of periodontal ligament-derived stem cells into retinal ganglion-like cells and its microRNA signature
Source: Sci Rep. 2015 Nov 9;5:16429. doi: 10.1038/srep16429 (PMC4637909; doi:10.1038/srep16429)
Supplement: Supplementary Information [file srep16429-s1.doc]

**Transdifferentiation of periodontal ligament-derived stem cells into retinal ganglion-like cells and its microRNA signature**

Tsz Kin Ng,^1^ Jasmine S.Y. Yung,^1^ Kwong Wai Choy,^2^ Di Cao,^1^ Christopher K.S. Leung,^1^ Herman S. Cheung,^3,4,*^ Chi Pui Pang.^1,*^

^1^ Department of Ophthalmology & Visual Sciences, The Chinese University of Hong Kong, Hong Kong.

^2^ Department of Obstetrics & Gynaecology, The Chinese University of Hong Kong, Hong Kong, Hong Kong.

^3^ Geriatric Research, Education and Clinical Center, Miami Veterans Affairs Medical Center, Miami, FL, United States.

^4^ Department of Biomedical Engineering, College of Engineering, University of Miami, Coral Gables, FL, United States.

*** Correspondence**:

Prof. Chi Pui Pang

Department of Ophthalmology and Visual Sciences, The Chinese University of Hong Kong, Hong Kong Eye Hospital, 147K Argyle Street, Kowloon, Hong Kong

Email: cppang@cuhk.edu.hk; Phone: +852-39435801; Fax: +852-27159490

Prof. Herman S. Cheung

Miami VA Medical Center, 1201 NW 16^th^ Street, Miami, FL 33125

Phone: +1-(305)-575-3388; Fax: +1-(305)-575-3365

Email: hcheung@med.maimi.edu

**Supplementary table 1: Primers for gene expression analysis.**

| **Gene** |  | **Primer Sequence (5' > 3')** | **Tm (℃)** | **Accession number** |
| --- | --- | --- | --- | --- |
| *GAPDH* | *F:* | TGTTGCCATCAATGACCCCTT | 60 | NM_002046.3 |
|  | *R:* | CTCCACGACGTACTCAGCG |  |  |
| *PAX6* | *F:* | CCTCATTTCCCGCTCTGGTT | 60 | NM_000280.4 |
|  | *R:* | TCTCAGATTCCTATGCTGATTGGT |  |  |
| *VSX2* | *F:* | CTACTGGGGATGCACAAAAAGTC | 60 | NM_182894.2 |
|  | *R:* | GCTGTTCTCCCTCAGTTCCTC |  |  |
| *ATOH7* | *F:* | TGAAAGGCCTTCCTCGAACC | 60 | NM_145178.3 |
|  | *R:* | CGGCCTTCTGTTCTACTGGA |  |  |
| *POU4F2* | *F:* | TCGTCCGCTCTTTTCTCTCC | 60 | NM_004575.2 |
|  | *R:* | CTCAGTTGTTCGCTCGTTCG |  |  |
| *GJA1* | *F:* | AAGTACCAAACAGCAGCGGA | 60 | NM_000165.3 |
|  | *R:* | TGGGCACCACTCTTTTGCTTA |  |  |
| *KLF4* | *F:* | CACATTAATGAGGCAGCCACC | 60 | NM_004235.4 |
|  | *R:* | GGAAGTCGCTTCATGTGGGA |  |  |
| *MAP3K12* | *F:* | TTCACCATCATACCAGGGGC | 60 | NM_006301.3 |
|  | *R:* | ATGTAGCTGCAGGACATGGG |  |  |
| *PTEN* | *F:* | TAAGGACCAGAGACAAAAAGGGAG | 60 | NM_000314.4 |
|  | *R:* | TCCGCCACTGAACATTGGAA |  |  |
| *SOCS3* | *F:* | AGAGTCCTGTAGCTCTGGGG | 60 | NM_003955.3 |
|  | *R:* | TGTGGTTGCTATCGTCCCAC |  |  |
| *VEGFA* | *F:* | CATCACCATGCAGATTATGCGG | 60 | NM_003376.5 |
|  | *R:* | CCGGGATTTCTTGCGCTTTC |  |  |
| *Pax6* | *F:* | GAATTCTGCAGGTGTCCAACG | 60 | NM_013001.2 |
|  | *R:* | TGTCTCGGATTTCCCAAGCAA |  |  |
| *Brn3a* | *F:* | GCGGACTTTGCGAGTGTTTT | 60 | XM_006252434.1 |
|  | *R:* | CCAGGCTAGCGAAGAGGTTG |  |  |
| *Brn3b* | *F:* | CTGAGCACAACTTTGCCGTG | 60 | NM_134355.1 |
|  | *R:* | GCCTGCTTGCTATTCAGGGA |  |  |
| *Rho* | *F:* | ACGTCACCGTACAGCACAAG | 60 | NM_033441.1 |
|  | *R:* | CCGATTTCACCTCCAAGGGTG |  |  |
| *Gfap* | *F:* | ATTGCTGGAGGGCGAAGAAA | 60 | NM_017009.2 |
|  | *R:* | TCTCCACCGTCTTTACCACGA |  |  |

**Supplementary table 2: Antibodies for protein expression analysis.**

| **Protein** | **Company** | **Catalog number** | **Source** | **Dilution factor** |
| --- | --- | --- | --- | --- |
| PAX6 | Covance | PRB-278P | rabbit | 1:500 |
| CHX10 | Santa Cruz Biotechnology | sc-21690 | goat | 1:500 |
| MAP2 | Upstate Biotechnology | 05-346 | mouse | 1:1000 |
| NEUROD1 | Abcam | ab60704 | mouse | 1:100 |
| POU4F2 | Abcam | ab56026 | rabbit | 1:500 |
| TAU | Upstate Biotechnology | 05-838 | mouse | 1:1000 |
| SIX3 | Sigma-Aldrich | SAB1412238 | mouse | 1:100 |
| β-III tubulin | Covance | MMS-435P | mouse | 1:1000 |
| Synaptophysin | Santa Cruz Biotechnology | sc-12737 | mouse | 1:100 |
| PTEN | Cell Signaling Technology | 9559 | mouse | 1:500 |
| VEGF | Upstate Biotechnology | 05-443 | mouse | 1:500 |
| SOCS3 | Cell Signaling Technology | 2923 | rabbit | 1:500 |
| CUX1 | Proteintech | 11733-1-AP | rabbit | 1:500 |
| β-ACTIN | Sigma-Aldrich | A3854 | mouse | 1:2000 |

**Supplementary table 3: Predicted target gene list for eye development.**

| **Gene** | **Description** | **miRNA** |
| --- | --- | --- |
| *TWSG1* | twisted gastrulation homolog 1 (Drosophila) | *hsa-miR-136* |
| *YY1* | YY1 transcription factor | *hsa-miR-410* |
| *MITF* | microphthalmia-associated transcription factor | *hsa-miR-1825* |
| *RPGRIP1L* | RPGRIP1-like | *hsa-miR-27b* |
| *NHS* | Nance-Horan syndrome | *hsa-miR-148a* |
| *VSX1* | visual system homeobox 1 | *hsa-miR-20b* |
| *COL5A2* | collagen, type V, alpha 2 | *hsa-miR-29b* |
| *PROX1* | prospero homeobox 1 | *hsa-miR-181a* |
| *GLI3* | GLI-Kruppel family member GLI3 | *hsa-miR-7; hsa-miR-143* |
| *FOXP2* | forkhead box P2 | *hsa-miR-134* |
| *MFN2* | mitofusin 2 | *hsa-miR-20b* |
| *CHD7* | chromodomain helicase DNA binding protein 7 | *hsa-miR-410* |
| *PVRL1* | poliovirus receptor-related 1 | *hsa-miR-1305* |
| *SP3* | Sp3 transcription factor | *hsa-miR-487a; hsa-miR-410* |
| *BCL11B* | B-cell CLL/lymphoma 11B (zinc finger protein) | *hsa-miR-1825* |
| *MAP3K1* | mitogen-activated protein kinase kinase kinase 1 | *hsa-miR-1225-3p; hsa-let-7i; hsa-miR-18a* |
| ***VEGFA*** | vascular endothelial growth factor A | *hsa-miR-29b; hsa-miR-15b* |
| *MAB21L1* | mab-21-like 1 (C. elegans) | *hsa-miR-199a-5p* |
| ***KLF4*** | Kruppel-like factor 4 (gut) | *hsa-miR-7* |

**Supplementary table 4: Predicted target gene list for neuron differentiation**

| **Gene** | **Description** | **miRNA** |
| --- | --- | --- |
| *ALS2* | amyotrophic lateral sclerosis 2 (juvenile) | *hsa-miR-199a-5p* |
| *NRP1* | neuropilin 1 | *hsa-miR-148a* |
| *SEPT2* | septin 2 | *hsa-miR-15b* |
| *AGTPBP1* | ATP/GTP binding protein 1 | *hsa-miR-1305* |
| *WNT3A* | wingless-type MMTV integration site family, member 3A | *hsa-miR-15b* |
| ***GJA1*** | gap junction protein, alpha 1, 43kDa | *hsa-miR-301a* |
| *PAX3* | paired box 3 | *hsa-miR-199a-5p* |
| ***PTEN*** | phosphatase and tensin homolog | *hsa-miR-148a* |
| *GLI3* | GLI-Kruppel family member GLI3 | *hsa-miR-7; hsa-miR-143* |
| *NRCAM* | neuronal cell adhesion molecule | *hsa-miR-874* |
| *WNT1* | wingless-type MMTV integration site family, member 1 | *hsa-miR-148a* |
| *ZFP91* | zinc finger protein 91 homolog (mouse) | *hsa-miR-1305* |
| *BDNF* | brain-derived neurotrophic factor | *hsa-miR-134; hsa-miR-933* |
| *HOXC8* | homeobox C8 | *hsa-miR-148a* |
| *S1PR1* | sphingosine-1-phosphate receptor 1 | *hsa-miR-181a; hsa-miR-148a* |
| *ANK3* | ankyrin 3, node of Ranvier (ankyrin G) | *hsa-miR-199a-5p* |
| *ATXN10* | ataxin 10 | *hsa-miR-128* |
| *ROBO1* | roundabout, axon guidance receptor, homolog 1 | *hsa-miR-29b* |
| *NR2F6* | nuclear receptor subfamily 2, group F, member 6 | *hsa-miR-27b* |
| *CNTNAP2* | contactin associated protein-like 2 | *hsa-miR-874* |
| *CDH23* | cadherin-like 23 | *hsa-miR-296-5p* |
| *RET* | ret proto-oncogene | *hsa-miR-128* |
| *STX3* | syntaxin 3 | *hsa-let-7i* |
| *PTPRR* | protein tyrosine phosphatase, receptor type, R | *hsa-miR-15b* |
| *TBR1* | T-box, brain, 1 | *hsa-miR-542-5p* |
| *HOXC10* | homeobox C10 | *hsa-miR-136* |
| *ASCL1* | achaete-scute complex homolog 1 (Drosophila) | *hsa-miR-374b* |
| *SLITRK1* | SLIT and NTRK-like family, member 1 | *hsa-miR-27b* |
| *NDEL1* | nudE nuclear distribution gene E homolog (A. nidulans)-like 1 | *hsa-miR-575* |
| *ADM* | adrenomedullin | *hsa-miR-410* |
| *SEMA4F* | semaphorin 4F | *hsa-let-7i* |
| ***VEGFA*** | vascular endothelial growth factor A | *hsa-miR-29b; hsa-miR-15b* |
| *CNTN4* | contactin 4 | *hsa-miR-181a; hsa-miR-148a* |
| *SIAH1* | seven in absentia homolog 1 (Drosophila) | *hsa-miR-1305* |
| *RELN* | reelin | *hsa-miR-128* |
| *CDK5R1* | cyclin-dependent kinase 5, regulatory subunit 1 (p35) | *hsa-miR-148a* |
| *DRD1* | dopamine receptor D1 | *hsa-miR-20b* |
| *LPPR4* | plasticity related gene 1 | *hsa-miR-30d* |
| *ERBB3* | v-erb-b2 erythroblastic leukemia viral oncogene homolog 3 | *hsa-miR-148a* |
| *ONECUT2* | one cut homeobox 2 | *hsa-miR-199a-5p* |
| *SOX5* | SRY (sex determining region Y)-box 5 | *hsa-miR-485-5p; hsa-miR-132* |
| *DSCAML1* | Down syndrome cell adhesion molecule like 1 | *hsa-miR-376a; hsa-miR-376b* |
| *HPRT1* | hypoxanthine phosphoribosyltransferase 1 | *hsa-miR-301a* |
| *MYCBP2* | MYC binding protein 2 | *hsa-miR-181a; hsa-miR-132* |
| *PVRL1* | poliovirus receptor-related 1 | *hsa-miR-1305* |
| *BCL11B* | B-cell CLL/lymphoma 11B | *hsa-miR-1825* |
| *MTPN* | myotrophin | *hsa-miR-136* |
| *MTCH1* | mitochondrial carrier homolog 1 (C. elegans) | *hsa-miR-301a* |
| *NUMB* | numb homolog (Drosophila) | *hsa-miR-410* |
| *DCX* | doublecortex; lissencephaly, X-linked (doublecortin) | *hsa-miR-324-5p* |
| *LHX8* | LIM homeobox 8 | *hsa-miR-30d* |
| *ETV4* | ets variant gene 4 | *hsa-miR-361-3p* |
| *NKX2-2* | NK2 homeobox 2 | *hsa-miR-374b* |
| *DCLK1* | doublecortin-like kinase 1 | *hsa-miR-15b* |
| *CEBPB* | CCAAT/enhancer binding protein (C/EBP), beta | *hsa-miR-374b; hsa-miR-155* |
| *NTF3* | neurotrophin 3 | *hsa-miR-374b* |
| *MAP2K1* | mitogen-activated protein kinase kinase 1 | *hsa-miR-15b* |
| *MCF2* | MCF.2 cell line derived transforming sequence | *hsa-miR-143* |
| *TGFBR1* | transforming growth factor, beta receptor I | *hsa-let-7i* |
| *CREB1* | cAMP responsive element binding protein 1 | *hsa-miR-27b* |
| *FOXA1* | forkhead box A1 | *hsa-miR-132* |
| *NR4A2* | nuclear receptor subfamily 4, group A, member 2 | *hsa-miR-30d* |
| *NTN4* | netrin 4 | *hsa-miR-20b* |
| *NTNG1* | netrin G1 | *hsa-miR-132|hsa-miR-199a-5p* |
| *NTNG2* | netrin G2 | *hsa-miR-132|hsa-miR-199a-5p* |
| *EN1* | engrailed homeobox 1 | *hsa-miR-374b* |
| *NEUROG2* | neurogenin 2 | *hsa-miR-374b* |
| *DOCK7* | dedicator of cytokinesis 7 | *hsa-miR-30d* |
| *ISL1* | ISL LIM homeobox 1 | *hsa-miR-128* |
| *VSX1* | visual system homeobox 1 | *hsa-miR-20b* |
| *ATP7A* | ATPase, Cu++ transporting, alpha polypeptide | *hsa-miR-148a* |
| *EPHA4* | EPH receptor A4 | *hsa-miR-20b* |
| *EPHA7* | EPH receptor A7 | *hsa-miR-15b* |
| *NEDD4* | neural precursor cell expressed, developmentally down-regulated 4 | *hsa-miR-27b; hsa-miR-18a* |
| *ULK2* | unc-51-like kinase 2 (C. elegans) | *hsa-miR-301a* |
| *PBX3* | pre-B-cell leukemia homeobox 3 | *hsa-miR-20b* |
| *CACNA1A* | calcium channel, voltage-dependent, P/Q type, alpha 1A subunit | *hsa-miR-143* |


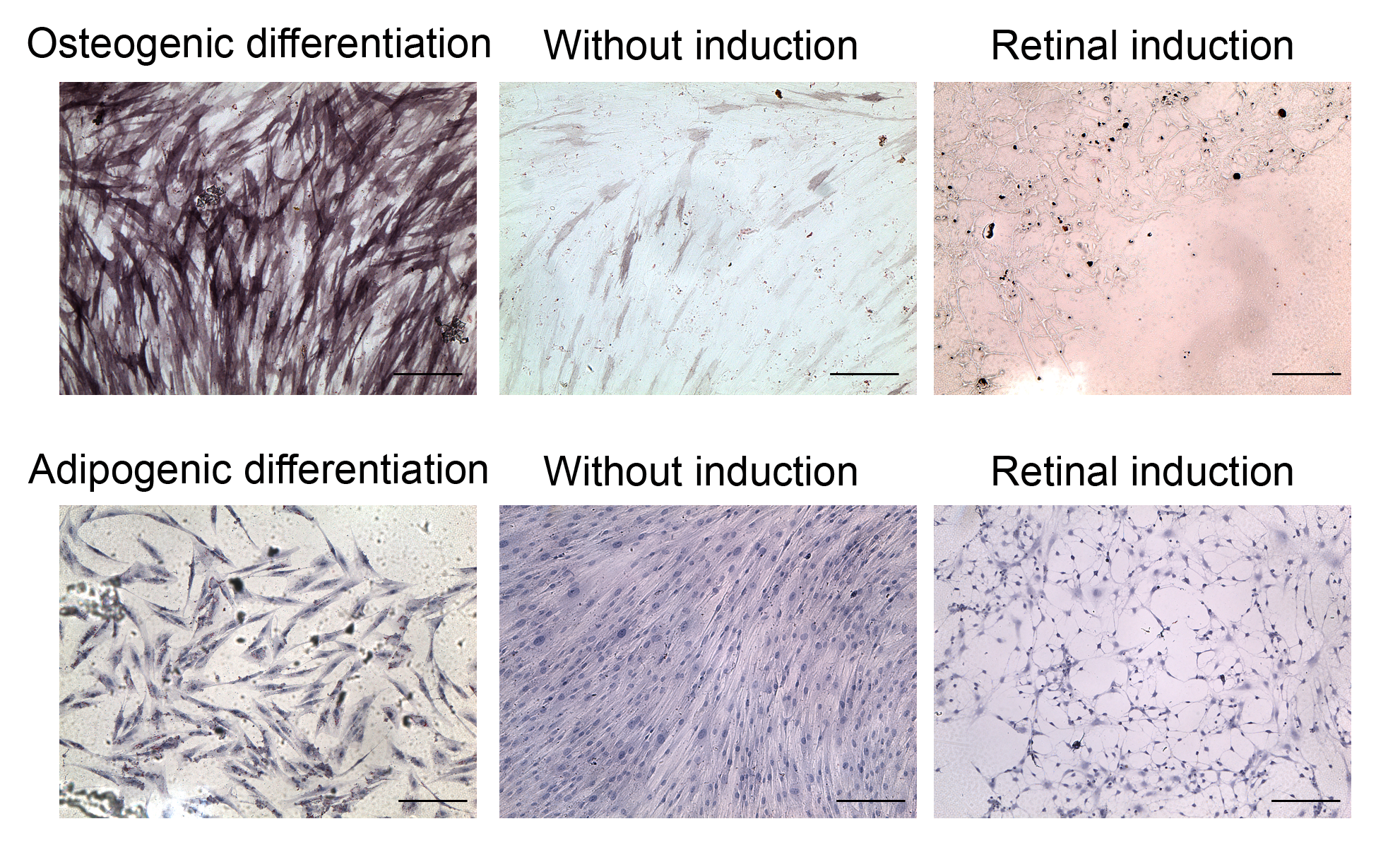


**Supplementary figure 1: Alkaline phosphatase and lipid analyses in retinal-induced PDLSCs.**

Human PDLSCs were treated under the retinal induction protocol for 24 days. Alkaline phosphatase activity was assessed by Burstone’s staining protocol with NBT/BCIP reagent, whereas lipid was evaluated by Oil Red O staining, and nuclei were counter-stained by hematoxylin. Scale bar: 200 μm.
